# Childcare disruptions and maternal health during the COVID-19 pandemic

**DOI:** 10.1093/haschl/qxae061

**Published:** 2024-05-21

**Authors:** Colleen L MacCallum-Bridges, Lindsay K Admon, Jamie R Daw

**Affiliations:** Department of Obstetrics and Gynecology, University of Michigan, Ann Arbor, MI 48109, United States; Department of Obstetrics and Gynecology, University of Michigan, Ann Arbor, MI 48109, United States; Department of Health Policy and Management, Columbia University Mailman School of Public Health, New York, NY 10032, United States

**Keywords:** maternal health, health equity, childcare, COVID-19 pandemic

## Abstract

During the COVID-19 pandemic, nearly all US states enacted stay-at-home orders, upending usual childcare arrangements and providing a unique opportunity to study the association between childcare disruptions and maternal health. Using data from the 2021–2022 National Survey of Children's Health, we estimated the association between childcare disruptions due to the COVID-19 pandemic and self-reported mental and physical health among female parents of young children (ages 0–5 years). Further, we assessed racial, ethnic, and socioeconomic disparities in (1) the prevalence of childcare disruptions due to the COVID-19 pandemic and (2) the association between childcare disruptions and mental or physical health. Female parents who experienced childcare disruptions due to the COVID-19 pandemic were less likely to report excellent or very good mental (−7.4 percentage points) or physical (−2.5 percentage points) health. Further, childcare disruptions were more common among parents with greater socioeconomic privilege (ie, higher education, higher income), but may have been more detrimental to health among parents with less socioeconomic privilege (eg, lower education, lower income, and single parents). As state and federal policymakers take action to address the maternal health crisis in the United States, our findings suggest that measures to improve childcare stability may also promote maternal health and health equity.

## Introduction

The United States (US) is currently experiencing a maternal health crisis. The maternal mortality rate in the US is more than twice as high as most other high-income countries and nearly doubled between 2018 and 2021, increasing from 17.4 to 32.9 deaths per 100 000 live births.^1,2^ There is also evidence that US mothers are struggling with their health beyond the perinatal and postpartum period. For example, there is evidence that parents, and mothers in particular, have lower levels of emotional well-being than non-parents.^3,4^ Numerous studies have also suggested that parental and maternal stress increased during the COVID-19 pandemic while mental health worsened.^5-11^ Additionally, the maternal health crisis is disproportionately borne by women and birthing people of color (particularly those who identify as Black or Indigenous), rural individuals, and people with lower income and less formal education.^1,2,12-14^ As a result, it has become a public health and policy priority to improve maternal health and health equity in the US.^15,16^

One potential determinant of maternal health that has been understudied, and is amenable to policy intervention, is access to stable and reliable childcare.^17,18^ Childcare is often cited as a major stressor for parents due to high cost, inadequate supply (ie, childcare deserts), and general instability (eg, high staff turnover rates, closures).^19-22^ Unreliable or precarious childcare can also impact parents’ labor participation and healthcare utilization.^19,23-27^ As a result, childcare has the potential to influence parental health through multiple pathways. In fact, previous studies have shown that early childcare precarity and difficulty arranging childcare are associated with greater psychological distress and worse mental health among mothers,^3,28,29^ while institutional supports for childcare have been associated with greater parental well-being.^4,30-32^

During the COVID-19 pandemic, nearly all US states enacted stay-at-home orders,^33^ upending usual childcare arrangements and forcing many parents—particularly mothers, who more often engage in primary caretaking^34^—to reduce their hours at work or withdraw from the workforce to address heightened childcare demands.^35-37^ As such, the COVID-19 pandemic provides a unique opportunity to study the association between childcare disruptions and maternal health. Small studies based on convenience samples have found preliminary evidence that pandemic-related disruptions to childcare were associated with worse mental health among female caregivers and postpartum individuals.^38,39^ There is also evidence that pandemic effects on stress and stressors differed by race, ethnicity, and socioeconomic status,^36,40^ but little work has been done to investigate whether childcare disruptions due to the COVID-19 pandemic affected racial, ethnic, or socioeconomic disparities in maternal health. A better understanding of the impacts of childcare stability on maternal health and health equity could help inform whether policymakers should consider childcare policies as potential interventions to improve maternal health and health equity.

The objectives of this study were to use nationally representative data to (1) estimate the association between childcare disruptions due to the COVID-19 pandemic and maternal mental and physical health and (2) explore the potential impact of such disruptions on racial, ethnic, and socioeconomic disparities in maternal health.

## Data and methods

### Data source and study population

We used cross-sectional data from the 2021 and 2022 National Survey of Children's Health (NSCH). The NSCH is conducted annually to collect information regarding key indicators of child health and well-being, parental or caregiver health, and social context (eg, family interactions, neighborhood environment, and school experiences).^41^ Using the US Census Bureau's Master Address File, the NSCH selects an address-based probabilistic sample of noninstitutionalized children aged 0–17 years from all 50 states and the District of Columbia, oversampling young children (0–5 years) and children with special health care needs.^41^ When sampling weights are applied, the NSCH sample is representative of noninstitutionalized US children ages 0–17 years.^41^ The overall response rate was 40.3% in 2021 and 39.1% in 2022, which is similar to response rates observed prior to the COVID-19 pandemic (eg, response rates ranged from 40.7% in 2016 to 42.4% in 2019).^41-44^ Additional details regarding NSCH survey methodology have been published elsewhere.^45^

From these data, we included female biological or adoptive parents of children aged 0–5 years (*n* = 35 654) (Appendix Figure A1; to access the Appendix, click on the details tab of the article online). We focus on parents of children ages 0–5 years because childcare needs are generally highest in early childhood, among infants and preschool-aged children (ie, ages 0–5 years).^46^ Moreover, parents of children ages 6–17 years were either not asked about childcare disruptions due to COVID-19 (ages 12–17 years), or the phrasing of the NSCH question was ambiguous regarding whether school closures should be considered a childcare disruption (ages 6–11 years),^47^ which was a major source of increased childcare need among this school-aged group. We identified female biological or adoptive parents—hereafter referred to as “female parents”—using 2 items on the NSCH: (1) caregiver sex and (2) caregiver relationship to the child. Among households with 2 female parents (*n* = 303; 0.8% of the analytic sample), we included the parent who provided answers to the survey (referred to as adult 1 in the NSCH documentation).

### Outcomes

We evaluated 2 primary maternal health outcomes: (1) mental health status, captured by the question “In general, how is your mental or emotional health?” and (2) physical health status, captured by the question “In general, how is your physical health?” Respondents could select excellent, very good, good, fair, or poor for each item. We dichotomized these options into 2 categories: excellent or very good health vs good, fair, or poor health. We chose this dichotomization for both empirical and conceptual reasons. First, poor health is rarely reported (eg, 1.1% of the analytic sample reported poor mental health and only 0.6% reported poor physical health). Moreover, we are particularly interested in thinking about ways to promote exceptional health (ie, better than good). For these reasons, we operationalized the outcome measures to indicate very good or excellent health.

### Exposure

The primary exposure was reporting a childcare disruption due to the COVID-19 pandemic, measured by the NSCH survey question, “During the past 12 months, has this child's regular daycare or other childcare arrangement been closed or unavailable at any time because of the coronavirus pandemic?”

### Covariates

We included characteristics of the child (age, race-ethnicity), female parent (age, education level), and household (family structure, income, metropolitan residence, state of residence) as covariates because we hypothesized these factors could confound the relationship between childcare disruption and maternal health. Covariates were operationalized using the categories presented in Table 1. Caregiver race-ethnicity is not captured in the NSCH. Thus, we included child race-ethnicity as an imperfect proxy for the family's exposure to structural racism.^48^ We also accounted for state-level factors that could potentially confound the hypothesized relationship by including state fixed-effects, state-year economic indicators (unemployment rate, poverty rate), and the duration of COVID-19–related stay-at-home orders (measured in months), which serves as a proxy for the intensity of the state's response to the COVID-19 pandemic. Definitions and data sources for state-year economic indicators and duration of COVID-19–related stay-at-home orders can be found in Appendix Table A1.

**Table 1.** Sample characteristics of the 2021–2022 NSCH analytic sample, overall and by exposure status (*n* = 35 654).

	Overall (*n* = 35 654)	Unexposed: childcare not disrupted by COVID-19 pandemic (*n* = 22 949)	Exposed: childcare disrupted by COVID-19 pandemic (*n* = 12 705)	*P* ^a^
Child characteristics				
Age (y)				<.0001
0	3371 (15.7)	2904 (19.5)	467 (6.1)	
1	4510 (15.9)	3152 (16.7)	1358 (14.1)	
2	7580 (16.7)	4883 (16.7)	2697 (16.8)	
3	6762 (17.2)	4204 (16.6)	2558 (18.9)	
4	6733 (17.8)	3841 (15.5)	2892 (23.5)	
5	6698 (16.7)	3965 (15.1)	2733 (20.7)	
Race-ethnicity				<.0001
Hispanic	4893 (25.4)	3467 (27.4)	1426 (20.4)	
NH AIAN	121 (0.4)	88 (0.4)	33 (0.4)	
NH Asian	1914 (4.6)	1317 (4.5)	597 (4.8)	
NH Black	1653 (10.4)	1101 (10.1)	552 (11.1)	
NH NHOPI	83 (0.2)	58 (0.2)	25 (0.1)	
NH White	24 157 (52.0)	15 147 (50.6)	9010 (55.5)	
NH multiple races	2833 (7.0)	1771 (6.8)	1062 (7.6)	
Female parent characteristics				
Age (y)				<.0001
<30	6392 (21.0)	4995 (24.4)	1397 (12.4)	
30–39	23 053 (62.3)	14 415 (60.8)	8638 (65.8)	
40–49	5990 (16.0)	3386 (13.9)	2604 (21.2)	
50+	156 (0.5)	106 (0.5)	50 (0.4)	
Missing	63 (0.3)	47 (0.4)	16 (0.1)	
Education level				<.0001
Less than high school	982 (9.1)	850 (10.9)	132 (4.7)	
High school	3634 (14.6)	2987 (17.0)	647 (8.8)	
Some college, associate degree, or vocational program	8425 (23.3)	6138 (24.9)	2287 (19.3)	
Bachelor's degree or more	22 613 (52.9)	12 974 (47.2)	9639 (67.2)	
Household characteristics				
Family structure				.1045
Two-parent household	31 461 (83.4)	20 066 (82.9)	11 395 (84.6)	
Single-parent household	4193 (16.6)	2883 (17.1)	1310 (15.4)	
Household income level				<.0001
<100% of the FPL	3767 (17.0)	2934 (19.3)	833 (11.1)	
100%–199% of the FPL	5112 (18.9)	3961 (21.1)	1151 (13.4)	
200%–299% of the FPL	5601 (16.1)	4072 (16.7)	1529 (14.5)	
300%–399% of the FPL	5148 (12.0)	3361 (12.1)	1787 (12.0)	
≥400% of the FPL	16 027 (36.0)	8621 (30.8)	7406 (49.0)	
Metropolitan residence				<.0001
Yes	26 357 (83.2)	16 900 (82.9)	9457 (84.0)	
No	5621 (11.6)	3926 (12.7)	1695 (8.7)	
Missing	3676 (5.2)	2123 (4.4)	1553 (7.3)	
Exposure				
Childcare disrupted by the COVID-19 pandemic	12 705 (28.5)	0 (0.0)	12 705 (100.0)	

Abbreviations: AIAN, American Indian Alaska Native; FPL, Federal Poverty Level; NH, non-Hispanic; NHOPI, Native Hawaiian or other Pacific Islander; NSCH, National Survey of Children's Health.

Data are presented as unweighted frequencies and weighted percentages.

^a^ *P* values correspond to overall *F* test in simple linear probability model.

### Statistical analysis

We described sample characteristics, overall and within exposed and unexposed groups, using frequencies and percentages. We used simple linear probability models to assess whether sample characteristics differed between unexposed and exposed groups. We evaluated the association between the exposure and each outcome using unadjusted and adjusted linear probability models, estimating the prevalence difference (PD) in excellent or very good health (mental or physical) between female parents who experienced a childcare disruption due to the COVID-19 pandemic and those who did not. In adjusted models, we included child age, child race-ethnicity, female parent age, female parent education level, family structure, household income level, metropolitan residence status, state of residence, state-year–level economic factors (unemployment rate, poverty rate), and duration of COVID-19–related stay-at-home orders.

We also conducted analyses to explore whether racial-ethnic and socioeconomic disparities in maternal health were affected by childcare disruptions due to COVID-19. Because health disparities can arise through disparities in the prevalence of an exposure and/or disparities in the impact of an exposure,^49^ we evaluated both pathways. To consider the first pathway, we used unadjusted linear probability models to estimate the PD in childcare disruption due to COVID-19 by race-ethnicity, maternal education level, family structure, and household income level. To consider the second pathway, we added 2-way interactions to our unadjusted and adjusted regression models to estimate the association between childcare disruption due to COVID-19 and physical and mental health by race-ethnicity, education level, family structure, and household income level. These interaction terms were also used to estimate whether the associations between childcare disruption and health differed by race-ethnicity, education level, family structure, or household income level. In both analyses, we considered the reference group to be the most socially or economically privileged group (ie, the non-Hispanic [NH] White group, parents with at least a bachelor's degree, 2-parent households, and households with income ≥400% of the Federal Poverty Level [FPL]).

To further contextualize the findings of these analyses, we also assessed disparities in childcare use and disparities in severity of childcare disruptions. We assessed disparities in childcare use because it provides information about who was using childcare, and therefore who was at risk of childcare disruption. To assess differences in childcare use, we estimated PDs for receiving regular childcare, defined as 10 or more hours of childcare per week from someone other than a parent or guardian. We also assessed disparities in the severity of childcare disruptions because the severity of disruption may influence health impacts (eg, more severe disruptions may be more harmful). To assess disparities in the severity of childcare disruptions, we estimated PDs for childcare-related work disruptions (ie, someone in the family quitting a job, not taking a job, or greatly changing their job because of problems with childcare) among respondents who reported a childcare disruption due to the COVID-19 pandemic.

We excluded respondents who were missing data on the outcome (*n* = 280) or exposure (*n* = 488) (Appendix Figure A1). Because missingness of covariates was relatively rare (0.3% missing parental age, 5.2% missing metropolitan residence), and multiple imputation is generally considered unnecessary when missingness is rare,^50^ we addressed missing covariate data by incorporating missing indicators (ie, a “missing” category) for these variables. All analyses were conducted in Stata/SE 18.0 (StataCorp, College Station, TX) using svy commands and NSCH-provided sampling weights to account for the sampling design of the NSCH and produce estimates that are nationally representative of US children. Because this study uses only publicly available data from the NSCH,^51^ this study does not qualify as human subjects research and was deemed exempt from review by the University of Michigan's Institutional Review Board (HUM00250409).

## Results

### Sample characteristics

Table 1 shows the child, parent, and household characteristics of the study sample overall and by exposure to a COVID-19–related childcare disruption. Just over half (52.0%) of included children were NH White, while 25.4% were Hispanic, 10.4% were NH Black, 7.0% reported multiple races, 4.6% were NH Asian, 0.4% were NH American Indian/Alaska Native, and 0.2% were NH Native Hawaiian or other Pacific Islander. Most female parents were 30–39 years old (62.3%), had at least a bachelor's degree (52.9%), reported a household income 200% or more of the FPL (64.0%), were part of a 2-parent household (83.4%), and lived in a metropolitan area (83.2%). Overall, 28.5% of children aged 0–5 years experienced a COVID-19–related childcare disruption. Children who experienced a childcare disruption were older (3–5 years) and more likely to be NH White than children who did not experience a childcare disruption. Additionally, female parents of children with a COVID-19–related childcare disruption were more likely to be aged 30–49 years, have indicators of higher socioeconomic status (at least a bachelor's degree, a household income ≥400% of the FPL), and live in a metropolitan area.

### Primary outcomes

The unadjusted prevalence of excellent or very good mental health was −4.5 percentage points (ppt) lower (95% CI: −6.7, −2.2) among female parents who experienced a childcare disruption due to the COVID-19 pandemic compared with those who did not (Table 2). After adjusting for child, female parent, and household characteristics, and state-level factors, the estimated difference increased to −7.4 ppt (95% CI: −9.7, −5.0) (Table 2). The unadjusted prevalence of excellent or very good physical health did not differ significantly between female parents who did and did not experience a childcare disruption due to COVID-19. However, while the unadjusted model showed no significant difference, childcare disruptions due to the COVID-19 pandemic were significantly associated with a lower prevalence of excellent or very good physical health based on the adjusted model (−2.5 ppt; 95% CI: −4.7, −0.3) (Table 2).

**Table 2.** Estimates for the association between childcare disruptions due to COVID-19 and excellent or very good mental or physical health among female parents of children aged 0–5 years, based on the 2021–2022 NSCH analytic sample (*n* = 35 654).

	Prevalence of outcome		Prevalence difference (PD)	
	Unexposed	Exposed	Unadjusted PD (95% CI)	Adjusted PD (95% CI)
Excellent or very good mental health	69.3%	64.8%	−4.5 (−6.7, −2.2)^a^	−7.4 (−9.7, −5.0)^a^
Excellent or very good physical health	72.9%	73.8%	0.8 (−1.3, 3.0)	−2.5 (−4.7, −0.3)^a^

Abbreviation: NSCH, National Survey of Children's Health.

The adjusted model includes child age, child race-ethnicity, female parent age group, female parent educational level, household family structure, household income, metropolitan residence, state of residence, state-year unemployment rate, state-year poverty rate, and length of COVID-19 stay-at-home order (in months). Prevalence difference is reported in percentage points.

^a^Indicates statistical significance at the .05 significance level.

### Racial-ethnic and socioeconomic disparities

**Figure 1.**
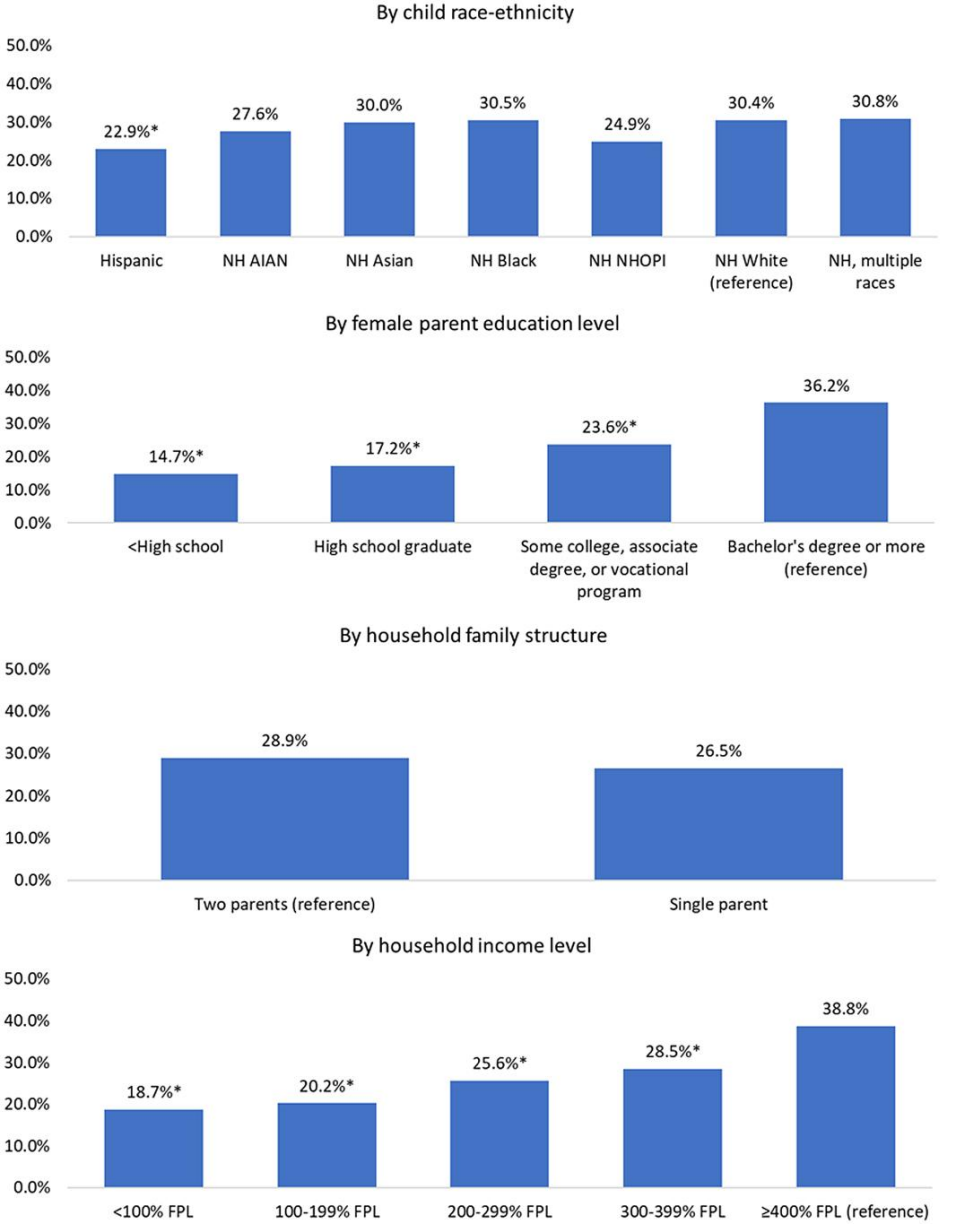
Prevalence of childcare disruptions due to COVID-19 among racial-ethnic and socioeconomic subgroups, based on the 2021–2022 National Survey of Children's Health (NSCH) analytic sample of female parents of children ages 0–5 years (*n* = 35 654). *Indicates a statistically significant difference at the .05 significance level compared with the reference group. Abbreviations: FPL, Federal Poverty Level; NH, non-Hispanic; NHOPI, Native Hawaiian or other Pacific Islander.

The prevalence of childcare disruption due to the COVID-19 pandemic differed by race-ethnicity, parental education, and household income, but not family structure (Figure 1). The prevalence of childcare disruptions was higher among NH White children (30.4%) compared with Hispanic children (22.9%) (*P* < .001) (Figure 1). The prevalence of childcare disruptions was also higher among female parents with at least a bachelor's degree (36.2%) compared with those with less education and for female parents in households with incomes 400% or more of the FPL (38.8%) compared with those with lower incomes. Similar patterns were observed when considering differences in regular childcare use (Appendix Figure A2). However, among female parents who experienced a childcare disruption due to the pandemic, racially minoritized (Hispanic, NH Black) and socioeconomically disadvantaged (lower education, lower income, single-parent) groups were more likely to report childcare-related work disruptions (Appendix Figure A3).

**Figure 2.**
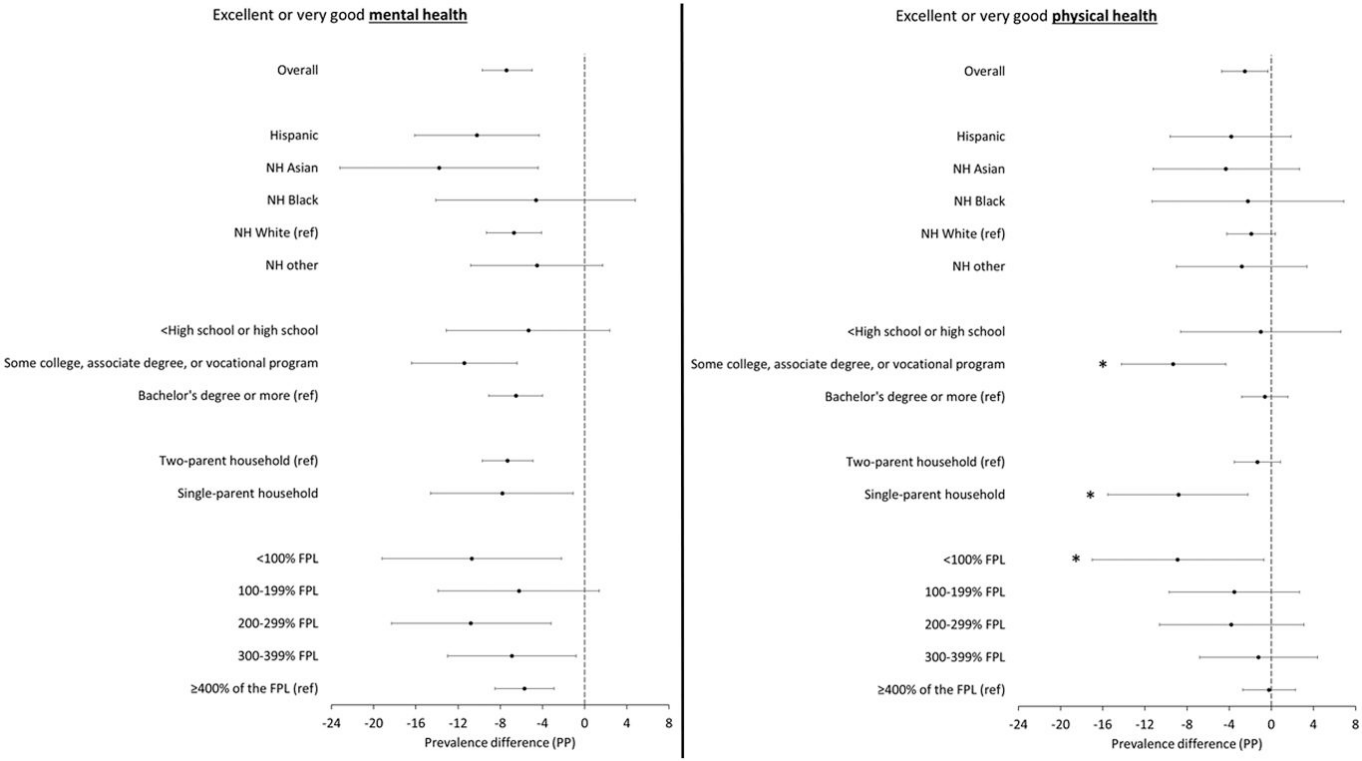
Adjusted estimates and 95% CIs for the association between childcare disruption due to the COVID-19 pandemic and maternal mental and physical health, overall and by sociodemographic subgroup, based on the 2021–2022 National Survey of Children's Health (NSCH) analytic sample of female parents of children ages 0–5 years (*n* = 35 564). The model adjusts for child age, child race-ethnicity, female parent age group, female parent education level, family structure, household income level, metropolitan residence, state of residence, state-year unemployment rate, state-year poverty rate, and length of COVID-19 stay-at-home order (in months). *Indicates that the prevalence difference among this group is statistically different from that of the reference group at the .05 significance level. The NH other racial-ethnic group includes children who were identified as NH American Indian/Alaska Native (*n* = 121), NH Native Hawaiian or other Pacific Islander (*n* = 83), or NH multiple races (*n* = 2833). Abbreviations: FPL, Federal Poverty Level; NH, non-Hispanic; PP, percentage points; ref, reference group.

We did not find statistically significant evidence that the association between childcare disruptions due to the COVID-19 pandemic and mental health differed by race-ethnicity, education, family structure, or income level among female parents (Figure 2; Appendix Table A2). The association with excellent or very good physical health, however, differed significantly by education, family structure, and household income level, but not by race-ethnicity. The association between childcare disruptions and excellent or very good physical health was significantly larger (more detrimental) among female parents who completed some college, an associate’s degree, or vocational training (−9.3 ppt; 95% CI: −14.2, −4.3) compared with those with at least a bachelor's degree (−0.6 ppt; 95% CI: −2.8, 1.6) (Figure 2; Appendix Table A3). Similarly, childcare disruptions were more negatively associated with excellent or very good physical health among households with incomes less than 100% of the FPL (−8.9 ppt; 95% CI: −17.0, −0.7) compared with households with incomes 400% or more of the FPL (−0.2 ppt; 95% CI: −2.7, 2.3), and among single-parent households (−8.8 ppt; 95% CI: −15.5, −2.2) compared with 2-parent households (−1.3 ppt; 95% CI: −3.5, 0.9) (Figure 2; Appendix Table A3).

## Discussion

Using a nationally representative sample of US children aged 0–5 years, we found evidence that childcare disruptions due to the COVID-19 pandemic were associated with worse mental and physical health among female parents. The prevalence of excellent or very good mental health was −7.4 ppt (or ∼10%) lower and the prevalence of excellent or very good physical health was −2.5 ppt (or ∼3%) lower among female parents who had a childcare disruption compared with those who did not. There was also evidence that childcare disruptions due to COVID-19 were less common, but perhaps more detrimental, among female parents with less social or economic privilege, at least with respect to physical health. COVID-19–related childcare disruptions were consistently associated with lower ratings of mental health among female parents in most of the examined racial-ethnic and socioeconomic subgroups, but the association with lower ratings of physical health was limited to female parents with lower education (those who had completed some college, an associate’s degree, or a vocational program), and those in lower income (incomes <100% of the FPL) or single-parent households.

Our finding that childcare disruptions due to the COVID-19 pandemic were negatively associated with mental and physical health is consistent with previous studies that found COVID-19–related stressors were associated with worse parental health.^10,38,39,52^ This finding is also consistent with previous work that found a lack of childcare, or precarious childcare, may prevent parents from utilizing health services and may be associated with worse parental health.^23-28^

We also found that childcare disruption due to the COVID-19 pandemic was more common among more socioeconomically advantaged groups, reflecting the fact that people with higher incomes and higher levels of education have greater access to and use of non-parental childcare.^46,53-55^ Importantly, we found that childcare disruptions—while less common among less advantaged socioeconomic groups—may have been more detrimental among less advantaged groups, particularly for the physical health of single parents and those with lower income and lower levels of education. This finding is consistent with Fundamental Cause Theory, which suggests that socioeconomic status acts as a root cause of health and health disparities by shaping access to resources that can mitigate risk and promote health (eg, resources such as money, power, prestige, and social connectedness).^56^ In alignment with this theory, more privileged parents would have more resources to draw upon and deploy to offset the adverse health effects of childcare disruptions during the COVID-19 pandemic, whereas less privileged parents would have fewer such resources (eg, employer-provided workplace accommodations like paid leave and work-time flexibility^57-59^; material resources for health aids^60^). It is curious, however, that the association between childcare disruptions and mental health did not differ significantly between sociodemographic subgroups. These null findings may be due, in part, to insufficient statistical power, given that the observed differences in associations for mental health were smaller, but in the same direction, as those observed for physical health. It is further possible that the resources available to more socioeconomically privileged parents are better-suited for preserving physical health than mental health, or that the effort required to draw upon and deploy these resources may act as its own source of stress,^22^ thereby limiting benefits for mental health.

Together, our findings suggest that childcare may play a role in shaping maternal health and health equity. Many federal, state, and local policymakers have made maternal health a priority, but policies that support parents in caring for young children are rarely discussed as a way to improve maternal health. For example, there have recently been policy initiatives to expand access to parental leave,^61^ establish universal pre-kindergarten programs,^62,63^ and subsidize costs of childcare for both childcare providers and families seeking childcare.^62,64-66^ The main purpose of these initiatives—which have been primarily framed as economic and educational support measures—is to address 2 barriers that are commonly encountered by families seeking childcare: (1) limited supply, which often results in lengthy waitlists for childcare, and (2) unaffordable costs.^19,20,46,67^ However, our findings support the idea that childcare policies may provide more than just economic and educational support; they may also promote maternal health and health equity.^22^

This work has several limitations. First, all measures are self-reported and retrospective, meaning they may be susceptible to recall bias. Additionally, we used self-reported measures of mental and physical health that are nonspecific (ie, we do not know what conditions may contribute to an individual rating their health as they do). Second, due to small sample sizes, we had limited statistical power to detect disparate impacts of childcare disruption on mental and physical health by racial, ethnic, and socioeconomic subgroups. Third, we were unable to control for time-varying factors related to state-level responses to the COVID-19 pandemic, which may have coincided with childcare disruptions and simultaneously influenced parental stress and health. Fourth, while we were able to adjust for some of the observed differences in the characteristics of those who experienced childcare disruptions and those who did not, we cannot account for unobserved differences between these 2 groups. Given that the exposed group generally had higher levels of education and income than the unexposed group, we would expect these differences to attenuate our associational estimates, thereby leading us to underestimate the impact of childcare disruptions on mental and physical health. Thus, our estimates should not be interpreted as causal effects. Additional research is needed to understand the causal impact of childcare stability on maternal health—for example, by examining the impact of policies that shape access to reliable, high-quality childcare (eg, the ARPA [American Rescue Plan Act] Childcare Stabilization funds). Finally, we were unable to disentangle the mechanisms through which childcare stability may be impacting parental health (eg, stress, health behaviors, barriers to healthcare utilization), including the mechanisms through which more socioeconomically privileged parents may mitigate health impacts of childcare disruption (eg, material resources for health aids), both of which should be priorities for future research.

## Conclusion

In a nationally representative sample of children aged 0–5 years, we found that childcare disruption due to the COVID-19 pandemic was associated with poorer mental and physical health among female parents. Further, we found that these disruptions were more common among more socioeconomically privileged female parents but may have been more detrimental among less privileged female parents. As state and federal policymakers take actions to address poor maternal health outcomes in the US, our results suggest that measures to improve childcare stability may be 1 important lever to improve physical and mental health among female parents with young children.

## Supplementary Material

qxae061_Supplementary_Data
