# Supplementary material for: Childcare disruptions and maternal health during the COVID-19 pandemic
Source: Health Aff Sch. 2024 May 21;2(5):qxae061. doi: 10.1093/haschl/qxae061 (PMC11108245; doi:10.1093/haschl/qxae061)
Supplement: qxae061_Supplementary_Data [file qxae061_supplementary_data.zip › Appendix (clean) 4-29-24.docx]

**Appendix**

Figure A1. Flowchart for the unweighted analytic sample of female parents of children aged 0-5 years in the National Survey of Children’s Health (NSCH), 2021-2022

**Exclusion Criterion**

2021-2022 NSCH respondents

n = 104,995

Respondents who did not report a female biological or adoptive parent in the household (n=13,625)

Female biological or adoptive parents

n = 91,370

Female biological or adoptive parents of children ages 6-17 (n=54,932)

Female parents of children ages 0-5

n = 36,438

Missing information on mental or physical health (n=280)

Female parents of children ages 0-5 with complete outcome data

n = 36,158

Missing information on COVID-19 related disruptions to childcare (n=488)

Female parents of children ages 0-5 with complete exposure data

n = 35,670

Missing covariate information on parental education level (n=16)*

Analytic sample

n = 35,654

*^*^ Because missingness was so rare for parental education level (n=16, 0.06%), we did not include a missing indicator (i.e., a “missing” category) for this covariate. Rather, we excluded the n=16 respondents with missing data for parental education level. For all other covariates with missingness (parental age and metropolitan residence), we used a missing indicator approach by including a “missing” category as shown in Exhibit 1.*

Table A1. Definitions and data sources for state-level factors

| **Covariate** | **Definition** | **Data Sources** |
| --- | --- | --- |
| Stay-at-home order | Indicates the duration (in months) for which a stay home order was in effect for each state. | COVID-19 US state policy database^1^ |
| State-year unemployment rate | The seasonally adjusted percent of the labor force that was unemployed. | US Bureau of Labor Statistics^2^ |
| State-year poverty rate | The percent of the state’s population that had an income below the federal poverty level in the past 12 months | American Community Survey^3,4^ |

References

1. Raifman J, Nocka K, Jones D, Bor J, Lipson S, Jay J, and Chan P. COVID-19 US state policy database. Accessed July 14, 2023. Available at: [https://github.com/USCOVIDpolicy/COVID-19-US-State-Policy-Database](https://github.com/USCOVIDpolicy/COVID-19-US-State-Policy-Database%20)
2. US Bureau of Labor Statistics. State unemployment rates over the last 10 years, seasonally adjusted. Accessed on July 12, 2023. Available at: <https://www.bls.gov/charts/state-employment-and-unemployment/state-unemployment-rates-animated.htm>
3. U.S. Census Bureau. "POVERTY STATUS IN THE PAST 12 MONTHS." American Community Survey, ACS 1-Year Estimates Subject Tables, Table S1701, 2021, <https://data.census.gov/table/ACSST1Y2021.S1701?q=poverty&g=010XX00US$0400000>. Accessed on April 12, 2024.
4. U.S. Census Bureau. "Poverty Status in the Past 12 Months." American Community Survey, ACS 1-Year Estimates Subject Tables, Table S1701, 2022, <https://data.census.gov/table/ACSST1Y2022.S1701?q=poverty&g=010XX00US$0400000>. Accessed on April 12, 2024.

Figure A2. Prevalence of regular childcare use among racial-ethnic and socioeconomic subgroups, based on the 2021-2022 NSCH analytic sample of female parents of children ages 0-5 (n=35,654)


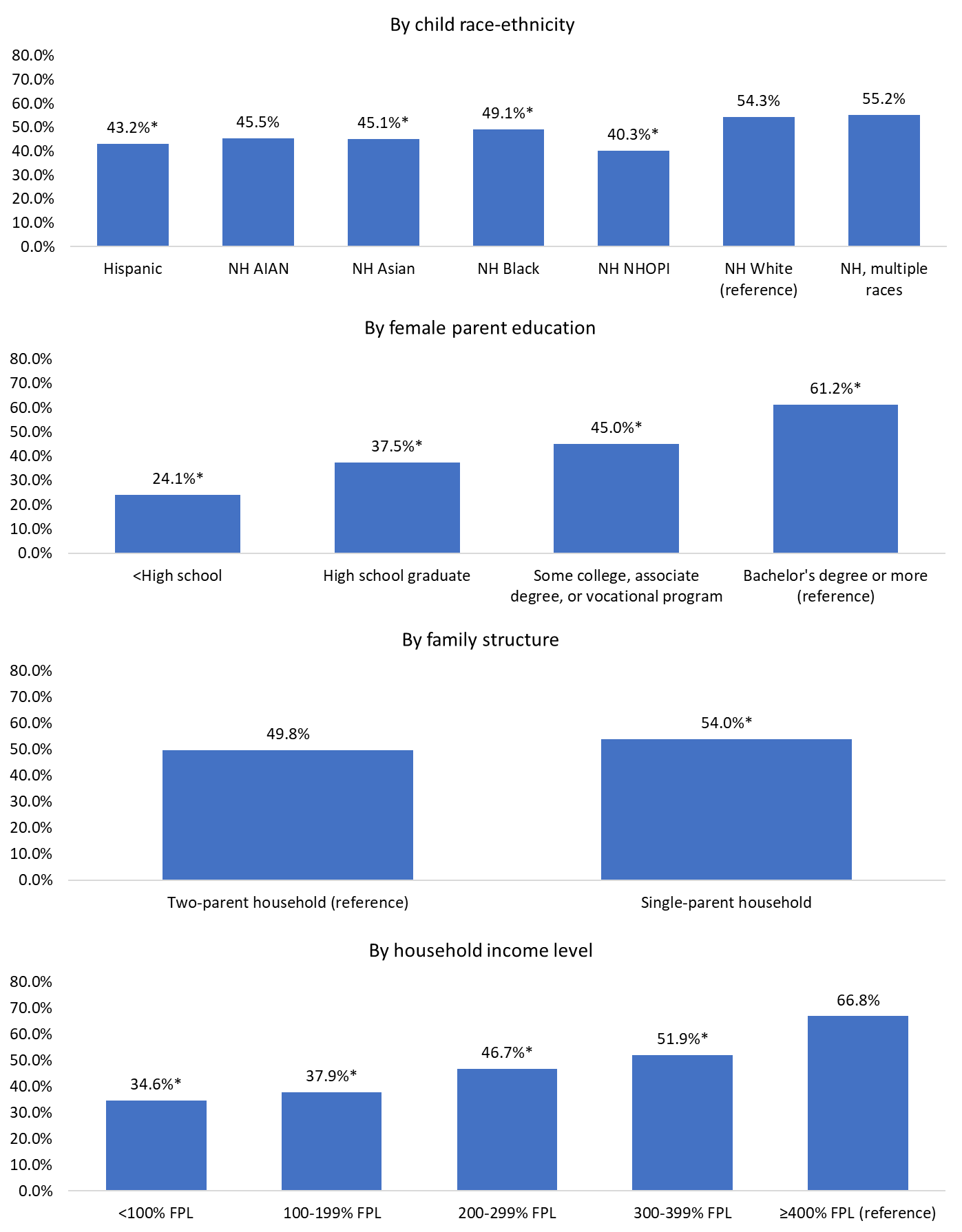


*NSCH = National Survey of Children’s Health. * Indicates a statistically significant difference at the 0.05 significance level compared to the reference group. “Regular childcare use” is defined as receiving ≥10 hours/week of childcare from someone other than their parent or guardian.*

Figure A3. Prevalence of childcare-related work disruptions among respondents who also reported a childcare disruption due to COVID-19, stratified by sociodemographic subgroup, based on the 2021-2022 NSCH analytic sample of female parents of children ages 0-5 (n=12,655)


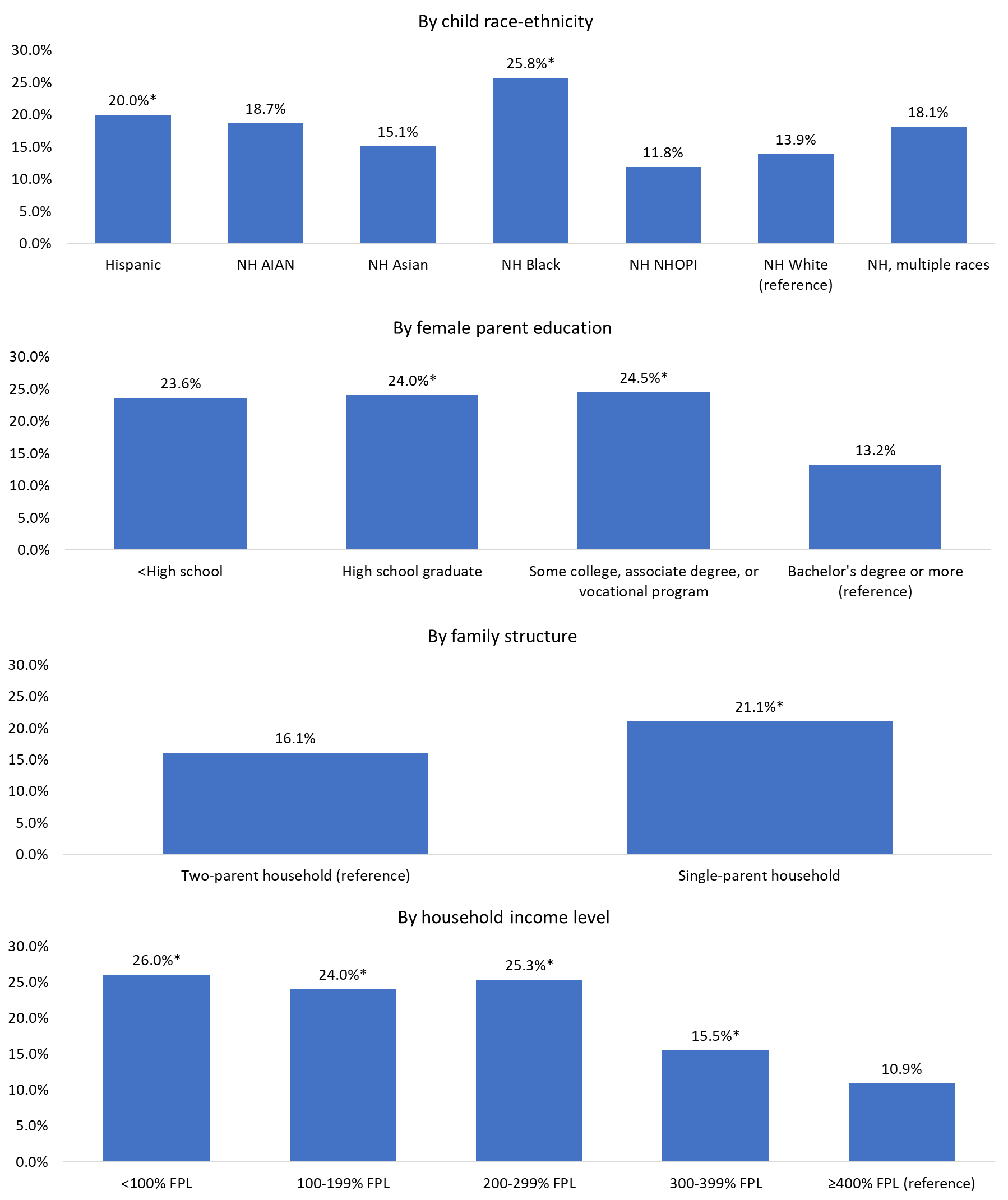


*NSCH = National Survey of Children’s Health. * Indicates a statistically significant difference at the 0.05 significance level compared to the reference group. Most privileged group used as reference group. Childcare-related work disruption identified through NSCH question that asked, “During the past 12 months, did you or anyone in the family have to quit a job, not take a job, or greatly change your job because of problems with childcare for this child?”*

Table A2. Prevalence difference estimates for the association between childcare disruptions due to COVID-19 and **excellent or very good mental health**, by sociodemographic subgroup, as estimated by prevalence differences based on the 2021-2022 NSCH analytic sample of female parents of children ages 0-5 (n=35,654)

|  | Unweighted sample size | Prevalence of outcome | | Prevalence difference (PD) | |
| --- | --- | --- | --- | --- | --- |
| Potential effect-modifier |  | Unexposed | Exposed | Unadjusted  PD (95% CI) | Adjusted  PD (95% CI) |
| Race-ethnicity |  |  |  |  |  |
| Hispanic | 4,893 | 69.2% | 62.0% | -7.2 (-13.2, -1.1)* | -10.2 (-16.1, -4.3)* |
| NH Asian | 1,914 | 83.9% | 74.7% | -9.2 (-18.2, -0.2)* | -13.8 (-23.2, -4.4)* |
| NH Black | 1,653 | 67.6% | 64.0% | -3.6 (-13.1, 5.9) | -4.6 (-14.1, 4.8) |
| NH White^a^ | 24,157 | 68.8% | 65.1% | -3.6 (-6.1, -1.1)* | -6.7 (-9.3, -4.1)* |
| NH other | 3,037 | 66.6% | 64.8% | -1.8 (-8.4, 4.8) | -4.5 (-10.8, 1.7) |
| Education level |  |  |  |  |  |
| <High school or high school graduate | 4,616 | 64.5% | 58.7% | -5.8 (-13.6, 2.0) | -5.3 (-13.1, 2.4) |
| Some college, associate degree, or vocational program | 8,425 | 64.8% | 53.8% | -10.9 (-16.0, -5.8)* | -11.4 (-16.4, -6.4)* |
| Bachelor’s degree or more^a^ | 22,613 | 74.5% | 69.2% | -5.3 (-7.8, -2.8)* | -6.5 (-9.1, -4.0)* |
| Family structure |  |  |  |  |  |
| Two-parent household^a^ | 31,461 | 70.3% | 66.2% | -4.1 (-6.5, -1.7)* | -7.3 (-9.7, -4.9)* |
| Single-parent household | 4,193 | 64.5% | 57.4% | -7.1 (-13.9, -0.2)* | -7.8 (-14.6, -1.1)* |
| Income level |  |  |  |  |  |
| <100% of the FPL | 3,767 | 62.4% | 51.5% | -10.8 (-19.6, -2.1)* | -10.7 (-19.2, -2.2)* |
| 100-199% of the FPL | 5,112 | 65.7% | 60.0% | -5.7 (-13.6, 2.2) | -6.2 (-13.9, 1.4) |
| 200-299% of the FPL | 5,601 | 66.7% | 56.7% | -9.9 (-17.4, -2.5)* | -10.8 (-18.3, -3.2)* |
| 300-399% of the FPL | 5,148 | 70.0% | 63.8% | -6.2 (-12.3, -0.2)* | -6.9 (-13, -0.8)* |
| ≥400% of the FPL^a^ | 16,027 | 77.2% | 71.8% | -5.4 (-8.2, -2.6)* | -5.7 (-8.5, -2.9)* |

*NSCH = National Survey of Children’s Health. *Indicates that the estimate is statistically different from the null value (0) at the 0.05 significance level. ^Indicates that the PD among this group is statistically different from that of the reference group at the 0.05 significance level. ^a^ Indicates the reference group. Adjusted model includes child age, child race-ethnicity, female parent age group, female parent education level, household family structure, household income, metropolitan residence, state of residence, state-year unemployment rate, state-year poverty rate, and length of COVID-19 stay-at-home order (in months). Sample sizes reported for each racial-ethnic or socioeconomic subgroup are unweighted, but prevalence and PD estimates are weighted. The NH other racial-ethnic group includes children who were identified as NH American Indian/Alaska Native (n=121), NH Native Hawaiian or other Pacific Islander (n=83), or NH multiple races (n=2,833).*

Table A3. Prevalence difference estimates for the association between childcare disruptions due to COVID-19 and **excellent or very good physical health**, by sociodemographic subgroup, as estimated by prevalence differences based on the 2021-2022 NSCH analytic sample of female parents of children ages 0-5 (n=35,654)

|  | Unweighted sample size | Prevalence of outcome | | Prevalence difference (PD) | |
| --- | --- | --- | --- | --- | --- |
| Potential effect-modifier |  | Unexposed | Exposed | Unadjusted  PD (95% CI) | Adjusted  PD (95% CI) |
| Race-ethnicity |  |  |  |  |  |
| Hispanic | 4,893 | 67.7% | 67.0% | -0.7 (-6.6, 5.1) | -3.8 (-9.6, 1.9) |
| NH Asian | 1,914 | 81.4% | 81.4% | 0.0 (-6.6, 6.7) | -4.3 (-11.2, 2.7) |
| NH Black | 1,653 | 68.0% | 66.3% | -1.7 (-10.9, 7.5) | -2.2 (-11.3, 6.9) |
| NH White^a^ | 24,157 | 76.5% | 77.8% | 1.3 (-0.9, 3.4) | -1.9 (-4.2, 0.4) |
| NH other | 3,037 | 69.5% | 69.4% | -0.1 (-6.8, 6.6) | -2.8 (-9.0, 3.4) |
| Education level |  |  |  |  |  |
| <High school or high school graduate | 4,616 | 62.6% | 59.8% | -2.7 (-10.4, 5.0) | -1.0 (-8.6, 6.6) |
| Some college, associate degree, or vocational program | 8,425 | 67.9% | 58.0% | -9.9 (-14.9, -4.8)*^ | -9.3 (-14.2, -4.3)*^ |
| Bachelor’s degree or more^a^ | 22,613 | 81.7% | 81.1% | -0.6 (-2.8, 1.5) | -0.6 (-2.8, 1.6) |
| Family structure |  |  |  |  |  |
| Two-parent household^a^ | 31,461 | 74.1% | 76.5% | 2.3 (0.2, 4.5)* | -1.3 (-3.5, 0.9) |
| Single-parent household | 4,193 | 67.1% | 58.9% | -8.1 (-15, -1.2)*^ | -8.8 (-15.5, -2.2)*^ |
| Income level |  |  |  |  |  |
| <100% of the FPL | 3,767 | 62.4% | 52.9% | -9.4 (-17.7, -1.2)*^ | -8.9 (-17.0, -0.7)*^ |
| 100-199% of the FPL | 5,112 | 68.2% | 64.9% | -3.3 (-9.8, 3.1) | -3.5 (-9.7, 2.7) |
| 200-299% of the FPL | 5,601 | 71.2% | 67.7% | -3.5 (-10.4, 3.3) | -3.8 (-10.6, 3.1) |
| 300-399% of the FPL | 5,148 | 76.2% | 75.3% | -0.9 (-6.5, 4.8) | -1.2 (-6.8, 4.4) |
| ≥400% of the FPL^a^ | 16,027 | 82.4% | 82.4% | -0.1 (-2.6, 2.5) | -0.2 (-2.7, 2.3) |

*NSCH = National Survey of Children’s Health. *Indicates that the estimate is statistically different from the null value (0) at the 0.05 significance level. ^Indicates that the PD among this group is statistically different from that of the reference group at the 0.05 significance level. ^a^ Indicates the reference group. Adjusted model includes child age, child race-ethnicity, female parent age group, female parent education level, household family structure, household income, metropolitan residence, state of residence, state-year unemployment rate, state-year poverty rate, and length of COVID-19 stay-at-home order (in months). Sample sizes reported for each racial-ethnic or socioeconomic subgroup are unweighted, but prevalence and PD estimates are weighted. The NH other racial-ethnic group includes children who were identified as NH American Indian/Alaska Native (n=121), NH Native Hawaiian or other Pacific Islander (n=83), or NH multiple races (n=2,833).*
